# Inhibition of IRE1α-mediated XBP1 mRNA cleavage by XBP1 reveals a novel regulatory process during the unfolded protein response

**DOI:** 10.12688/wellcomeopenres.11764.2

**Published:** 2017-10-09

**Authors:** Fiona Chalmers, Marcel van Lith, Bernadette Sweeney, Katharine Cain, Neil J. Bulleid

**Affiliations:** 1 Institute of Molecular, Cellular and Systems Biology, University of Glasgow, Glasgow, G12 8QQ, UK; 2 UCB Pharma Slough, Slough, SL1 3WE, UK

**Keywords:** Unfolded protein response, IRE1α, XBP1, ER stress response.

## Abstract

**Background**: The mammalian endoplasmic reticulum (ER) continuously adapts to the cellular secretory load by the activation of an unfolded protein response (UPR).  This stress response results in expansion of the ER, upregulation of proteins involved in protein folding and degradation, and attenuation of protein synthesis.  The response is orchestrated by three signalling pathways each activated by a specific signal transducer, either inositol requiring enzyme α (IRE1α), double-stranded RNA-activated protein kinase-like ER kinase (PERK) or activating transcription factor 6 (ATF6).  Activation of IRE1α results in its oligomerisation, autophosphorylation and stimulation of its ribonuclease activity.  The ribonuclease initiates the splicing of an intron from mRNA encoding the transcription factor, X-box binding protein 1 (XBP1), as well as degradation of specific mRNAs and microRNAs.

**Methods**: To investigate the consequence of expression of exogenous XBP1, we generated a stable cell-line expressing spliced XBP1 mRNA under the control of an inducible promotor.

**Results**: Following induction of expression, high levels of XBP1 protein were detected, which allowed upregulation of target genes in the absence of induction of the UPR.  Remarkably under stress conditions, the expression of exogenous XBP1 repressed splicing of endogenous XBP1 mRNA without repressing the activation of PERK.

**Conclusions**: These results illustrate that a feedback mechanism exists to attenuate Ire1α ribonuclease activity in the presence of XBP1.

## Introduction

The endoplasmic reticulum (ER) is the site of protein folding and post-translational modification of secreted and transmembrane proteins ^1^. Under stress conditions such as glucose starvation or a viral infection, the folding capacity of the ER can become compromised, leading to a potentially lethal build-up of unfolded or misfolded proteins ^2^. Protein folding homeostasis can be restored by triggering of a stress response called the unfolded protein response (UPR) ^3^. This complex and tightly-regulated process has downstream effects that enable the ER to adapt to stress conditions, and if this pro-survival strategy does not successfully restore ER homeostasis then pro-apoptotic signalling is induced ^4^.

The mammalian UPR is formed from three distinct but overlapping signalling branches, each governed by an initial effector protein localised to the ER membrane. These proteins are activating transcription factor 6 (ATF6), protein kinase-like ER kinase (PERK) and inositol requiring enzyme α (IRE1α), and are activated in the presence of a build-up of incorrectly folded proteins ^5^. Of these three mammalian UPR effectors, IRE1α is the most conserved with its yeast homolog being solely responsible for the UPR in lower eukaryotes ^6^. The activation of its cytosolic endoribonuclease (RNase) domain enables processing of unspliced X-box binding protein 1 (XBP1) mRNA (XBP1u). Spliced transcripts (XBP1s) are translated into the protein XBP1 ^S^, a transcription factor that upregulates the expression of proteins involved in ER protein folding, ER associated degradation (ERAD) and lipid biogenesis as part of a concerted effort to increase the capacity of the ER to cope with unfolded proteins.

In addition to its RNase domain, IRE1α also contains a cytosolic kinase domain and a lumenal domain that senses ER stress, and these domains are connected by a single transmembrane domain ^7^. Upon activation, IRE1α forms dimers ^8^, via ‘face-to-face’ interactions ^9^. The ‘face-to-face’ dimer displays no RNase activity and represents an early stage in IRE1α activation; its main purpose is to bring the kinase domains into proximity to enable transautophosphorylation. Phosphorylation induces a change in structure into a ‘back-to-back’ dimer ^9^, which brings the RNase domains into direct contact, forming a functional RNase active site capable of splicing XBP1u.

Activated IRE1α is also able to digest mRNAs ^10^ and miRNAs ^11^ during a process termed Regulated IRE1α Dependent Decay (RIDD). It has been suggested that the specificity of IRE1α changes during the UPR, initially cleaving XBP1u, but during prolonged stress switching to the cleavage of mRNA coding for proteins upregulated during the UPR. The consequence is an exacerbation of the stress leading to apoptosis ^12^. In addition, the cleavage of miRNAs responsible for the downregulation of caspase-2 results in elevated levels of this protease and induction of apoptosis through the BAX/BAK-dependent pathway ^11^. What regulates this switch in specificity is unknown, but could be related to subtle changes in IRE1α phosphorylation status, conformation or interaction with IRE1α regulators ^13^.

Given the potential for IRE1α to activate proapoptotic factors during prolonged ER stress, it is important to understand how IRE1α activity is attenuated. Previous studies indicate that this attenuation may be the result of multiple mechanisms to reduce IRE1α protein, reverse oligomerisation or alter phosphorylation status. For example, IRE1α transcripts can be degraded by RIDD ^14^ and activated IRE1α dimers can be dephosphorylated by the phosphatase PP2Ce ^15^. In addition, the oxidation of thiols within the IRE1α lumenal domain occurs during activation, a modification that is reversed during IRE1α attenuation. This mechanism of attenuation is dependent upon oxidoreductase activity provided by P5, a member of the protein disulfide isomerase family ^16^. Finally the depletion of XBP1 ^S^ can be facilitated by XBP1 ^U^, the protein translated from XBP1u transcripts, and involves the binding of XBP1 ^U^ to XBP1 ^S^ and subsequent trafficking to the 26S proteasome for degradation ^17^.

In order to explore the regulatory mechanisms of IRE1α, we investigated the impact of high levels of expression of XBP1 ^S^ on the activity of IRE1α. Our results show that an abundance of XBP1 ^S^ represses endogenous XBP1 splicing during unstressed and stress conditions. This repression may represent a regulatory mechanism, where persistent ER stress attenuates IRE1α RNase activity at least towards XBP1 mRNA.

## Materials and methods

### Generation of stable cell lines

CHO-S X was generated by transfecting 4 μg of pTetOne vector (Clontech), containing cDNA for the human XBP1s sequence, into CHO-S cells (Life Technologies), co-transfected with 200ng of a linear selectable marker for puromycin (a vector:marker ratio of 20:1), with 4.2 μl of the transfection reagent NovaCHOice ( *Novagen*). Transfected cells and untransfected control cells were grown in a 6 cm diameter dish in adherent culture, and after 24 h of growth were trypsinised and 1/10 of the cells were transferred to a 15 cm dish and grown in 20 ml medium containing 12.5 μg/ml puromycin. The transfected cells were grown for approximately 10 days, refreshing the selection medium every 3–4 days. Colonies were identified and removed from the dish using trypsin-soaked cloning discs and transferred into the wells of a 12 well plate, with one colony per well. The clones were grown under selection for another 3–5 days until the well was confluent, then the surviving clones were transferred into T25 flasks and later T75 flasks. To generate the CHO-S XB cell line, CHO-S X cells were transfected with a BFP construct ^18^ using the same method as described above. The construct contained a G418 resistance gene, so the linear selectable marker was not required. Transfected cells were maintained under the dual selection of both 12.5 μg/mL puromycin and 2 mg/mL G418 (Promega) to maintain the BFP construct. Successful integration of the gene of interest was confirmed by western blotting.

### Maintenance of cell lines

CHO-S and CHO-S XB cells were grown in DMEM supplemented with 10% foetal bovine serum (FBS), 2mM glutamine, and non-essential amino acids at a working concentration of 10 μM for each amino acid (Gibco). Cells were grown as an adherent culture and split every 3–4 days using a standard trypsin protocol.

### XBP1 splicing assay

RNA was extracted from stress treated cells using Trizol Reagent (Ambion), following the manufacturer’s recommended protocol. RT-PCR was either carried out using the AccessQuick RT-PCR kit (Promega) or first strand cDNA was synthesised using Superscript II Reverse Transcriptase (Invitrogen) with oligo dTs (Invitrogen), according to the manufacturer’s specifications. cDNA for endogenous XBP1 was amplified using primers designed using CLC Genomics Workbench (v6) (RRID:SCR_011853) to be specific to the Chinese hamster XBP1 sequence, (5’-CGCTTGGGAATGGATG-3’ and 5’- CAGGGTCCAACTTGTCC-3’; Sigma-Aldrich). The PCR reaction yielded a 247 bp fragment for XBP1u and a 215 bp fragment for XBP1s, plus a hybrid band of approximately 280 bp following electrophoresis through a 2% agarose gel. Both endogenous and exogenous XBP1 were amplified simultaneously with a second, less specific set of primers, which can anneal to either the Chinese hamster or the human XBP1 sequence, 5’- ACAGCGCTTGGGGATGGATG-3’ and 5’- TGACTGGGTCCAAGTTGTCC-3’ (Sigma-Aldrich). PCR using these primers yielded the same fragments as the previous primer set, but with the addition of a fragment of 221 bp for exogenous XBP1s. Primers used for actin were 5’-CCACACCTTCTACAATGAGC-3’ and 5’-ACTCCTGCTTGCTGATCCAC-3’. PCR was performed with Accuzyme DNA polymerase (Bioline) with an initial melting step of 95°C for 5 min; then 35 cycles of 95°C for 45 s, an annealing step for 45 s, and 72°C for 45 s; followed by a final elongation step of 72°C for 10 min. The endogenous only primers used an annealing temperature of 60°C and the exogenous/endogenous primers used 62°C. For quantification, samples were separated on a 10% TBE polyacrylamide gel (BioRad) and analysed using Image J (v1.51q): RRID:SCR_003070.

### Cell lysis

After removing culture medium from the 6 cm diameter dish, the cells were washed with 20 mM NEM in PBS for 10 min. This was removed and 120 μl lysis buffer (50 mM Tris-HCl buffer containing 150 mM NaCl, 5 mM EDTA, 1% Triton X-100, 4mM NaF) was added to the monolayer and the cells were scraped into the buffer. This suspension was left on ice for 10 min, centrifuged at maximum speed for 10 min and the supernatant was extracted.

### SDS PAGE

Crude lysates were mixed with 0.2 M Tris-HCl (pH 6.8) containing 10% (w/v) SDS, 20% (v/v) glycerol and 0.05% (w/v) bromophenol blue (sample buffer) in a 4:1 ratio of lysate to sample buffer. Dithiothreitol (DTT) was added as a reducing agent at a working concentration of 20 mM. Polyacrylamide gels were loaded with 20–30 μl of this sample mixture and run at 20 mA per gel.

### Western blot

After separation, the samples were transferred to a nitrocellulose membrane (GE Healthcare) by wet transfer for 1 h at 250 mA using 25 mM Tris-HCl containing 200 mM glycine, 3.5 mM SDS and 20% (v/v) methanol. The blots were blocked in 5% (w/v) non-fat milk powder (Marvel) in 50 mM Tris-HCl buffer (pH 7.5) containing 150 mM NaCl and 0.1% (v/v) Tween (TBST) for 1 h. Primary antibodies were diluted in TBST and incubated overnight. Washes were performed three times for 10 min in TBST. Secondary antibodies were diluted in TBST and incubated for 1 h, and the blots shielded from light throughout the incubation. Blots were developed using the Odyssey SA scanner (Licor). The following primary antibodies were used: rabbit polyclonal anti-PDI ^19^, 1:500; rabbit polyclonal anti-XBP1 ^S ^(Biolegend, Cat# 619502, RRID:AB_315907), 1:500; rabbit monoclonal anti-PERK, (Cell Signalling, Cat# 3192 RRID:AB_2095847), 1:1000; mouse monoclonal anti-GAPDH, (Ambion, Cat# AM4300, RRID:AB_437392), 1:10000; rabbit polyclonal anti-actin, (Sigma-Aldrich Cat# A2103, RRID:AB_476694), 1:500. The following secondary antibodies were used: donkey polyclonal anti-mouse 680RD, (LI-COR Biosciences Cat# 926-68072, RRID:AB_10953628) 1:10000; donkey polyclonal anti-mouse 800CW (LI-COR Biosciences Cat# 926-32212, RRID:AB_621847), 1:10000; donkey polyclonal anti-rabbit 680RD, (LI-COR Biosciences Cat# 926-68073, RRID:AB_10954442), 1:10000; donkey polyclonal anti-rabbit 800CW (LI-COR Biosciences Cat# 926-32213, RRID:AB_621848), 1:10000.

### ER Tracker treatment

ER Tracker Green BODIPY FL Glibenclamide (Molecular Probes) was dissolved in DMSO to a 1 mM stock concentration. Treated cells were stained with 250 nM ER Tracker in Hank’s Balanced Salt Solution (HBSS) for 30 min, then trypsinised and resuspended in DMEM.

### ER stress treatments

Dithiothreitol (DTT) (Melford Labs) was prepared as 1 M stock in water and used at a 2.5 mM working concentration. Thapsigargin (Sigma-Aldrich) was prepared as a 2 mM stock in DMSO and used at 4 µM. Tunicamycin (Sigma-Aldrich) was prepared as a 10 mg/mL stock in DMSO and used at 10 µg/mL. PERK inhibitor (Tocris) was prepared as a 10mM stock in DMSO and used at 2.5 µM. Treatments were added for 3 h to cells that had been pretreated with doxycycline for 48 h.

### Flow cytometry

Cells were washed once in HBSS then run on a FACS Canto II flow cytometer (BD Biosciences) in HBSS. ER Tracker Green has an excitation at 504 nm and emission at 511 nm, which can be detected using the FITC filter on the flow cytometer. The data obtained was analysed using Flowing Software v2.5.1 (Turku Bioimaging).

## Results

**Figure 1.**
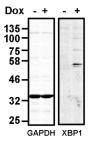
Induction of XBP1S expression in CHO-S XB cells. Western blot of lysates from CHO-S XB cells either uninduced or induced with doxycycline (Dox) for 3 days, probed with anti-XBP1s and anti-GAPDH as indicated. The blot is representative of the results obtained from three separate experiments.

To investigate the consequence of overexpression of human XBP1s in CHO-S cells, we created a stable cell-line expressing XBP1s mRNA under the control of a doxycycline inducible promoter. No XBP1 ^S^ was detected unless doxycycline was included in the medium ( Figure 1). Such tight regulation of expression allowed us to evaluate the effect of overexpression of XBP1s in the same cell line simply by culturing cells in the presence or absence of doxycycline. Interestingly additional bands at about 70 and 100 kDa can be observed which are specific to the induced sample. Similar species have been reported and are thought to represent XBP1 ^S^ that has undergone SUMOylation ^20^.

**Figure 2.**
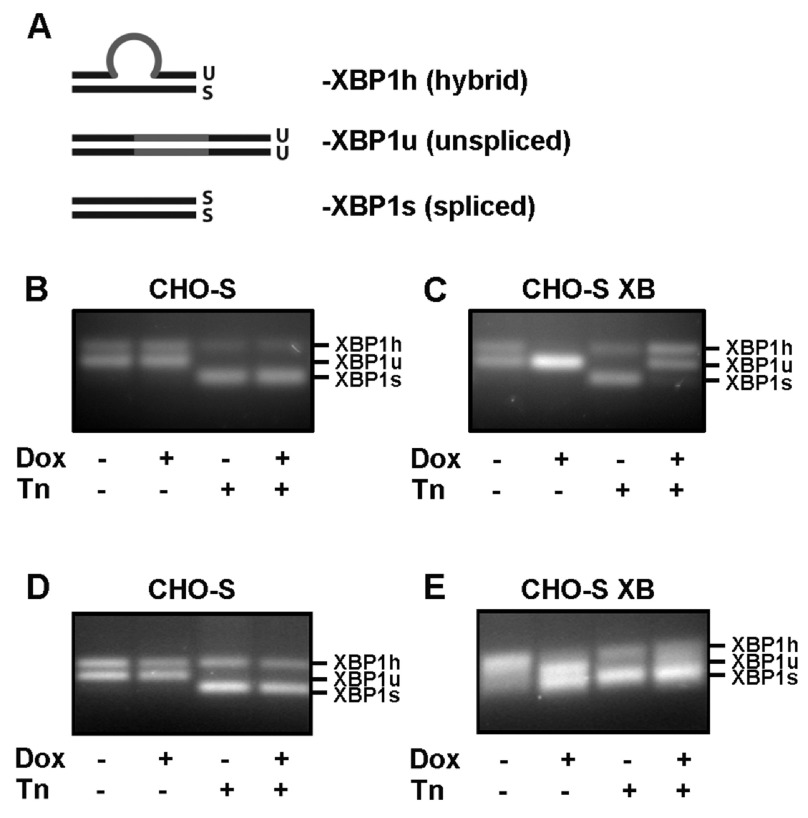
Consequence of XBP1s expression on XBP1u splicing in cell lines following endoplasmic reticulum stress. ( **A**) Schematic of the three possible cDNA products generated by primers that flank the XBP1 spliced intron (indicated in grey). Single stranded cDNA can anneal to either its complementary strand to generate double stranded XBP1u and XBP1s products, or generate a hybrid, XBP1h, formed from one strand XBP1u and one strand XBP1s. Of these three PCR products, XBP1s migrates the furthest on an agarose gel, followed by XBP1u and then by XBP1h, leading to the appearance of three distinct DNA bands. Adapted from 21. ( **B**– **E**) XBP1 splice assay of cDNA extracted from either ( **B**, **D**) CHO-S or ( **C**, **E**) CHO-S XB cells treated with doxycycline (Dox; for 3 days) and/or tunicamycin (Tn; for 3 h) as indicated. PCR reactions contained primers that anneal only to the endogenous CHO XBP1 sequence ( **B**, **C**) or primers that anneal to both the endogenous sequence and the exogenous XBP1s transcripts ( **D**, **E**). The experiment was performed at least three times, data from a representative experiment is shown.

To study the activation of IRE1α RNase activity, we assessed the cleavage of XBP1u mRNA before and after induction of human XBP1s expression, using an RT-PCR assay ^21^. By designing primers that flank the XBP1 spliced intron, cDNA derived from XBP1s and XBP1u transcripts can be amplified by PCR and distinguished from each other by a subtle, 26bp difference in product size when run on an agarose gel. This assay is also known to generate a third PCR product, shown diagrammatically ( Figure 2A), which is thought to be a hybrid double-stranded cDNA product consisting of one strand XBP1s and one strand XBP1u. This hybrid product migrates above double-stranded XBP1u on an agarose gel due to its bulkier structure ^21^. Two sets of primers were designed for the assay: the first set was designed to only anneal to the endogenous hamster sequence of XBP1, and these primers were used to quantify only endogenous XBP1s and XBP1u. The second set was designed to bind to both forms of endogenous CHO XBP1, and also to the exogenous human XBP1s transcript, allowing visualisation of all forms of XBP1 present in the cell.

In the absence of cell-stress, the endogenous XBP1 mRNA in CHO-S cells was present as a mixture of unspliced and hybrid forms ( Figures 2B and D). This result is consistent with a basal level of UPR signalling reported to be active under normal physiological conditions ^22^. As expected, there was no change in the splicing pattern following treatment with doxycycline, indicating that this chemical alone does not induce the UPR. Following treatment with the ER stress inducer tunicamycin, all of the XBP1 mRNA was converted to either the spliced or hybrid form indicative of a strong UPR ( Figures 2B and D). The splicing pattern of endogenous XBP1 mRNA in CHO-S XB cells in the absence of doxycycline, with or without cell stress was similar to that in CHO-S cells ( Figure 2C). However, in the absence of cell stress, incubation with doxycycline to induce XBP1s expression prevented any splicing of endogenous XBP1 mRNA, as evidenced by the absence of the hybrid form. Doxycycline induced expression of exogenous XBP1s, as seen by the presence of XBP1s when primers recognising both the human and hamster XBP1 were used in the assay ( Figure 2E). We consistently observed an increase in expression of XBP1u following induction of XBP1s expression, indicating the upregulation of XBP1u expression by XBP1s, as shown previously ^21, 23^. Under conditions of ER stress XBP1 mRNA was efficiently spliced in the absence of doxycycline, but this splicing was dramatically repressed after doxycycline treatment ( Figure 2C). This result indicates that XBP1 splicing by IRE1α is largely prevented in cells overexpressing XBP1s.

**Figure 3.**
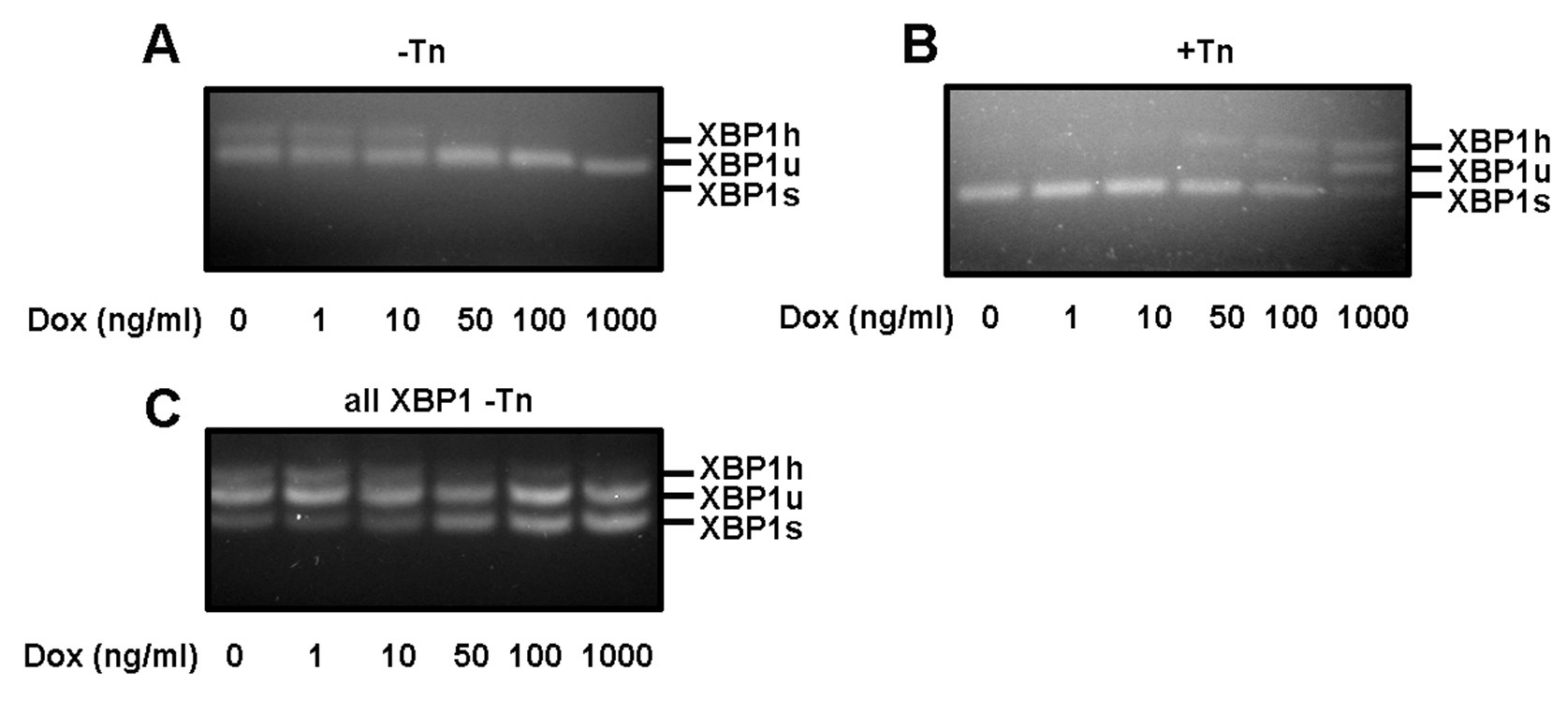
Inhibition of XBP1 splicing correlates with the amount of XBP1S. XBP1 splice assay of cDNA from CHO-S XB cells induced for 3 days with a range of doxycycline (Dox) concentrations, either not treated with tunicamycin (Tn) ( **A**, **C**), or treated with 10µg/mL Tn for 3 h. Primers specific for endogenous XBP1 ( **A**, **B**) or endogenous and exogenous XBP1 ( **C**) were used. The experiment was performed twice, data from a representative experiment is shown.

To determine whether there was a correlation between the induction of expression of exogenous XBP1s and the repression of IRE1α cleavage of endogenous XBP1 mRNA, we titrated the amount of added doxycycline to induce increasing amounts of exogenous XBP1s. The effect on cleavage of endogenous XBP1 mRNA became apparent after treating with 50 ng/ml of doxycycline both in the absence or presence of tunicamycin-induced ER stress ( Figures 3A and B). The effect increased with increasing concentrations of doxycycline with the greatest repression being most apparent at 1000 ng/ml in the presence of tunicamycin. When the presence of exogenously expressed XBP1s was evaluated using primers that amplify endogenous and exogenous XBP1, a clear increase in the XBP1s signal was observed at 50 ng/ml, which increased in intensity up to the highest concentration of doxycycline used ( Figure 3C). These results show a clear correlation between XBP1s expression and the repression of IRE1α-mediated cleavage of endogenous XBP1.

**Figure 4.**
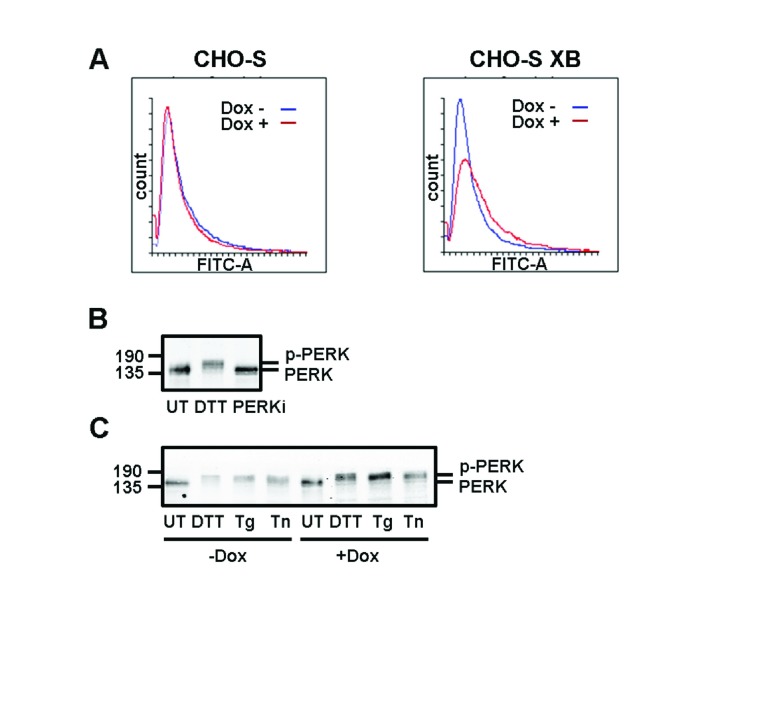
PERK activation is unchanged by overexpression of XBP1S. ( **A**) Flow cytometry analysis of fluorescence from CHO-S and CHO-S XB cells stained with fluorescent ER Tracker dye. Samples were either treated with doxycycline (Dox) for 3 days (red) or left untreated (blue). ( **B**) Western blot of lysates from CHO-S XB cells that were either untreated (UT), treated with a reducing agent (DTT) or with an inhibitor of PERK kinase activity (PERKi). Blots were probed with anti-PERK to display the extent of PERK phosphorylation. ( **C**) Anti-PERK western blot of CHO-S XB cells induced with Dox and subsequently treated with DTT, thapsigargin (Tg) or tunicamycin (Tn). Experiment ( **A**, **B** and **C**) were performed twice.

The expression of exogenous XBP1s should lead to the up-regulation of a number of proteins that are known to alleviate ER stress. Hence, the expression of XBP1s could prevent or suppress the tunicamycin-mediated activation of IRE1α, thereby repressing its RNase activity. To determine the consequence of XBP1s expression on ER expansion, we stained cells with ER Tracker, a dye that binds to potassium channels prominent at the ER membrane ^24^. Green fluorescence per cell was seen to increase following doxycycline treatment in CHO-S XB but not CHO-S cells, as quantified by FACS analysis ( Figure 4A). This result indicates that the expression of exogenous XBP1 does indeed cause an expansion in the ER, as seen previously when XBP1s is overexpressed in CHO cells ^25^. To determine whether XBP1s expression leads to a suppression of other branches of the UPR, we evaluated the activation of PERK, indicated by autophosphorylation. PERK phosphorylation was assayed by a shift in electrophoretic mobility to a slower migrating form after UPR induction, exemplified after treatment with DTT or the presence of a PERK kinase inhibitor ^26^ ( Figure 4B). There was no effect on PERK phosphorylation after treatment with a variety of UPR inducers (DTT, thapsigargin or tunicamycin) in the presence or absence of doxycycline in CHO-S XB cells. This result indicates that there is still a robust UPR activated following treatment with tunicamycin in cells overexpressing XBP1s as judged by PERK phosphorylation.

**Figure 5.**
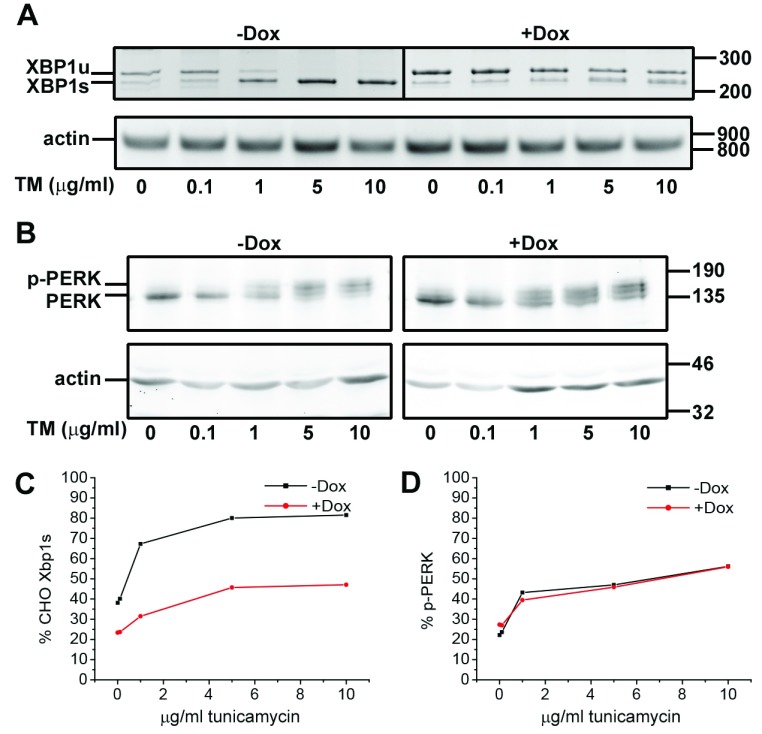
Differential effect of PERK and Ire1 activation is not due to differences in sensitivity to UPR induction. ( **A**) Expression of spliced exogenous XBP1 in CHO XB cells was induced by incubation with 1μg/ml doxycycline for 48 hours (refreshed after 24 hours). The cells were incubated with a tunicamycin (TM) concentration range as indicated for three hours. The samples were analysed for unspliced (XBP1u) and spliced (XBP1s) by RT-PCR, with actin as loading control (markers indicated as base pairs on the right). ( **B**) In a separate experiment, induced and uninduced CHO XB cells were treated with the same tunicamycin concentration range as in ( **A**) for three hours and analysed by SDS-PAGE and Western blot with an anti-PERK and anti-actin antibody (molecular weight markers indicated in kDa on the right). ( **C** and **D**) Quantification of spliced CHO XBP1 and phospho-PERK of ( **A**) and ( **B**), respectively. The results in **C**) and **D**) are from a single experiment. The experiments in **A**) and **B**) were carried out three times with consistent results.

To further evaluate the relative effect of exogenous XBP1s expression on Ire1α or PERK function we monitored their activation over a range of tunicamycin concentrations ( Figure 5). To allow more accurate quantification of endogenous XBP1 splicing we separated the PCR products by PAGE gels allowing a clear separation of the spliced and unspliced forms ( Figure 5A). Following quantification we observed that endogenous XBP1 splicing was efficient in the absence of exogenous XBP1s expression reaching a maxima at concentrations above 5μg/ml tunicamycin. Splicing was dramatically repressed at all concentrations of tunicamycin when tested in the presence of exogenous XBP1s ( Figure 5C). Interestingly, while the response to the inducer was repressed the sensitivity was similar with splicing occurring at 1μg/ml tunicamycin in the absence or presence of exogenous XBP1s. PERK was almost completely activated at the lower concentrations of tunicamycin (1μg/ml) in the presence or absence of XBP1s expression with no differences either in the sensitivity or level of the response ( Figure 5B, D). This result demonstrates that the differential effect of UPR induction on IRE1/PERK activation is not due to differences in their sensitivity to the inducer, rather it suggests that overexpression of XBP1s suppresses the IRE1α response while not effecting PERK.

## Discussion

Activation of the IRE1α branch of the UPR can lead to a variety of outcomes based on regulation of its RNase activity ^27^. This activity is tightly controlled by a number of mechanisms, including the transcript and protein levels of IRE1α, changes to its quaternary structure and by its phosphorylation and redox state. Our results indicate an additional mechanism of IRE1α feedback regulation involving XBP1s, which is able to repress RNase activity towards XBP1u. This regulation was revealed upon overexpression of XBP1s and occurred in the absence and presence of ER stress.

Overexpression of XBP1s leads to high levels of expression of XBP1 ^S^ protein, to the expansion of the ER and an increased expression of secreted proteins ^25, 28^. Preconditioning the ER to stress by XBP1s expression could prevent IRE1α activation, thereby repressing splicing of endogenous XBP1u. However, we showed that robust activation of PERK still occurs in cells overexpressing XBP1s upon ER stress. In addition the effect of exogenous XBP1s expression was to suppress IRE1α activity rather than alter its sensitivity towards UPR induction. As the mechanism for PERK and IRE1α activation requires BiP dissociation it seems unlikely that the suppression of Ire1α is due to increased levels of BiP. Hence, activation of IRE1α should occur even in the presence of XBP1 ^s^. The repression of IRE1α ability to splice XBP1u is, therefore, most likely to occur downstream of its activation during stress conditions.

Activation of IRE1α leads to its phosphorylation, which has been shown to promote dimerisation of its cytosolic domain ^29^. This suggests that phosphorylation activates IRE1α, whereas a phosphatase could be responsible for attenuating IRE1α. One phosphatase, PP2Ce, has been suggested to perform this role ^15^; however, the promoter for this gene does not display the ERSE, ERSE-II or UPRE consensus sequences characteristic of genes upregulated by XBP1s ^30, 31^. Also, it has been shown that hyper-phosphorylation rather that dephosphorylation of yeast IRE1 is required to deactivate this protein ^32^. Nevertheless, the possibility remains that an XBP1s-inducible phosphatase could attenuate the activity of IRE1α during prolonged induction of the UPR.

Alternatively, XBP1 ^s^ could block the initial phosphorylation and dimerisation of IRE1α in order to reduce the overall intensity of IRE1α signalling. In support of this hypothesis, it was reported that XBP1 ^s^ works in complex with Sec63 and BiP to negatively regulate IRE1α autophosphorylation ^33^. A mouse Sec63 knockout cell line was shown to constitutively activate IRE1α phosphorylation, regardless of the presence of ER stress. Intriguingly, this study revealed that the overexpression of XBP1 ^s^ in the Sec63 knockout cell line was able to abolish the activation of IRE1α almost entirely, even in the presence of tunicamycin, indicating that Sec63 and XBP1 ^s^ work in concert to regulate IRE1α phosphorylation. However, this study did not examine the effect of XBP1 ^s^ overexpression on IRE1α activation in a cell line with physiological levels of Sec63; circumstances that would be closer to the conditions used in the results reported here.

The abundance of IRE1α can be modulated by proteosomal degradation initiated by ubiquitination by the E3 ubiquitin ligase synoviolin (SYVN1), otherwise known as the ERAD component HRD1 ^34^. The ubiquitination of BiP-bound IRE1α monomers by SYVN1 leads to its dislocation from the ER and degradation by the proteasome. Under normal physiological conditions, BiP-bound IRE1α is continually degraded by ERAD, but the detachment of BiP allows for IRE1α to bypass interaction with SYVN1 and undergo accumulation and activation ^35^. Like other components of ERAD, SYVN1 is upregulated by XBP1s so it can be assumed that CHO-S XB would display high levels of SYNV1. This could lead to a reduction in IRE1α protein in XBP1s overexpressing cells; however, as only BiP-bound IRE1α is targeted for ERAD it is only this inactive form that would be affected by XBP1s upregulation, and not activated dimers. However, there is precedent for a reduction in total IRE1α unrelated to proteasomal degradation in the presence of stress. Heat shock treatment was shown to deplete IRE1α in a range of mammalian cell lines in a manner that could not be blocked by a proteasome inhibitor ^36^. The UPR was activated in these cells, indicated by the presence of IRE1α phosphorylation and other UPR hallmarks, but the specific mechanism for the degradation of IRE1α could not be clarified, and was attributed to an unknown method of suppressing extreme UPR signalling. It is possible that this mechanism could be mediated via XBP1s.

While we have noted here that XBP1s overexpression repressed XBP1u splicing, we have not investigated whether there is any suppression or even activation of activity towards other RNA substrates. It has been shown previously that overexpression of XBP1s had no effect on cell viability under non-stress conditions ^12^. Hence, under these conditions there was no suppression or activation of IRE1α RNase activity towards substrates other than XBP1u. It remains to be determined whether under stress conditions and in the presence of excess XBP1s, repression of IRE1α RNase activity extends to all mRNAs not just XBP1u.

Dysregulated IRE1α is a known contributing factor to a number of diseases, including multiple myeloma ^37^, epithelial cancers ^38^, Parkinson’s disease ^39^ and inflammatory bowel disease ^40^. Hence, increased knowledge of the regulatory mechanisms controlling IRE1α activity will help in understanding the pathogenesis of these diseases, as well as improving any therapeutic intervention.

## Data availability

The data referenced by this article are under copyright with the following copyright statement: Copyright: © 2017 Chalmers F et al.

The uncropped western blots, agarose and PAGE gels, and the FACS files can be found on the Open Source Framework (DOI: 10.17605/OSF.IO/BGCDE; ^41^)
