# Supplementary material for: Inhibition of IRE1α-mediated XBP1 mRNA cleavage by XBP1 reveals a novel regulatory process during the unfolded protein response
Source: Wellcome Open Res. 2017 Oct 9;2:36. Originally published 2017 Jun 5. [Version 2] doi: 10.12688/wellcomeopenres.11764.2 (PMC5645705; doi:10.12688/wellcomeopenres.11764.2)
Supplement: Peer review history

# Peer review: Referee response for version 2

**DOI:** 10.21956/wellcomeopenres.13868.r26825

**Published:** 2017-10-17

**Authors:** Adam M. Benham

**Affiliations:** 1 Department of Biosciences, Durham University, Durham, UK

The authors have included new data (Figure 5 of the revised manuscript) and controls to strengthen the work and have provided an acceptable rebuttal to the points raised in review.

I have read this submission. I believe that I have an appropriate level of expertise to confirm that it is of an acceptable scientific standard.

# Peer review: Referee response for version 2

**DOI:** 10.21956/wellcomeopenres.13868.r26824

**Published:** 2017-10-09

**Authors:** Stefan J. Marciniak

**Affiliations:** 1 Cambridge Institute for Medical Research, University of Cambridge, Cambridge, UK

The authors have now performed the main experiment suggested in my review. This shows that expression of exogenous XBP1s suppresses the splicing of endogenous XBP1 mRNA without having a detectable effect on the activation of PERK. This answers my concern and so I am satisfied to recommend acceptance.

I have read this submission. I believe that I have an appropriate level of expertise to confirm that it is of an acceptable scientific standard.

# Peer review: Referee response for version 1

**DOI:** 10.21956/wellcomeopenres.12708.r23238

**Published:** 2017-06-26

**Authors:** Adam M. Benham

**Affiliations:** 1 Department of Biosciences, Durham University, Durham, UK

This is an interesting report suggesting that novel feedback mechanisms exist to control ER stress responses in mammalian cells. The finding that exogenous XBP1 can repress the splicing of endogenous XBP1 without repressing PERK is noteworthy and suggests ways that in future, manipulation of individual branches of the UPR pathway could be used to target protein misfolding in disease, or to harness the ER more efficiently for the production of recombinant proteins. The paper is clearly written and the interpretation of the results is sound, although there are some aspects of the work that should be followed up.

 In Figure 1 (lane 4), there appear to be weak protein bands induced at 75 kD and 100 kD that react with the XBP1 antisera. Since these bands do not appear in the non-induced lysates, they may be specific. It would be interesting, therefore, to determine XBP1 expression and antibody reactivity in CHO-S XB cells induced with doxycycline and then treated +/- ER stress.

 The authors state that “as the mechanism for PERK and Ire1 activation requires BiP dissociation it seems unlikely that the absence of Ire1activation is due to increased levels of BiP.” This assertion could be directly tested by examining what happens to BiP mRNA and protein levels in the overexpressing cells versus control cells (+/- ER stress).

 In Figure 4, the experiments have been performed only once and the results, whilst clear, cannot be analysed statistically and are therefore somewhat preliminary. I would like to see the experiments in Figure 4 repeated, including a loading/blotting control for Figure 4C. 

 In Figure 4A, the flow cytometry data suggesting that ER expansion has occurred in the CHOS-XB cells is indirect, as the intracellular distribution of the dye may be influenced by stress responses. Taking this work forward in the future, it will be important to quantitate ER expansion directly e.g. in an adherent cell line, where ER shape and size can be assessed by confocal or electron microscopy.

I have read this submission. I believe that I have an appropriate level of expertise to confirm that it is of an acceptable scientific standard, however I have significant reservations, as outlined above.

# Response

**Published:** 2017-06-27

**Authors:** Neil Bulleid

**Affiliations:** University of Glasgow, UK

We thank the referee for insightful comments.  We are currently carrying out further experimentation to address the concerns.

# Response

**Published:** 2017-09-28

**Authors:** Neil Bulleid

**Affiliations:** University of Glasgow, UK

We thank the reviewer for his comments. We have now submitted a revised version of the paper and address the reveiwer's comments below. 

1. The weak protein bands seen in figure 1 that have a slower modility than XBP1 are indeed only present in the induced sample and are identifed with the antibody as contaning XBP1. It has been show previously that XBP1 can be modified by SUMOylation so we presume that these additional protein bands are due to this modification. We include a sentence in the results to suggest this possibility.
2. The differential effect of exogenously expressed XBP1 has now been tested by determining the effect of a range of tunicamycin concentrations on XBP1 splicing or PERK activation. This additional experiment indicates that Ire1 is activated and as sensitive to UPR inducers in the presence of exogenous XBP1 as in its absence, but its splicing activity is dramatically repressed. This results does not address the consequence of BiP dissociation from Ire1 or PERK directly but it clarifies that there is a supression of activity rather than activation. 
3. We have now carried out a repeat of Figure 4C with a loading control. (see ORF (DOI: 10.17605/OSF.Io/BGCDE;)
4. We note the suggestion by the reviewer.

# Peer review: Referee response for version 1

**DOI:** 10.21956/wellcomeopenres.12708.r23240

**Published:** 2017-06-06

**Authors:** Stefan J. Marciniak

**Affiliations:** 1 Cambridge Institute for Medical Research, University of Cambridge, Cambridge, UK

Protein misfolding in the endoplasmic reticulum (ER), ER stress, activates an unfolded protein response (UPR) ^1^. This UPR defends the cell by attenuating protein synthesis, enhancing ER chaperone levels and activity, and increasing ER-associated protein degradation. Three signalling pathways make up the UPR, each initiated by a separate ER stress sensor, IRE1α, PERK, and ATF6. These differ in their downstream signalling and in their kinetics of activation and deactivation ^2^^,^^3^. Activated IRE1α initiates the splicing of the mRNA encoding XBP1 ^4^^-^^6^. Unspliced XBP1 mRNA encodes an inactive protein, XBP1u, while spliced XBP1 mRNA encodes an active transcription factor, XBP1s.  XBP1s activates genes that increase ER chaperone expression and cause expansion of the ER.

 In the study of Chalmers *et al*, cells were generated that express active XBP1s protein in response to treatment with doxycycline. This caused expansion of the ER, consistent with induction of the known gene expression programme of XBP1s. When forced to express exogenous XBP1s, these cells also showed reduced splicing of endogenous XBP1u when treated with tunicamycin, an inhibitor of glycosylation commonly used to induce ER stress. In contrast, activation of PERK in response to ER stress-inducing agents (DTT, tunicamycin, or thapsigargin) appeared to remain unaffected.

 This is a well-written paper. The proposed direct feedback mechanism linking XBP1s to inhibition of XBP1u splicing would be an important finding if it could be demonstrated definitively; however, the current version of the paper leaves a second (less interesting) mechanism still to be excluded.

**Main concern**

 The authors suggest that XBP1s selectively blocks splicing of endogenous XBP1u, and that this does not involve a simple generalised resistance of the ER to stress. The authors acknowledge the second possibility in their discussion, but dismiss it because *(i)* PERK activation persists following induction of XBP1s and *(ii)* PERK and IRE1α are known to share a similar mechanism of activation ^7^. However, differences in the sensitivity of IRE1α and PERK to ER stress could account for their observations. If PERK were to be more sensitive than IRE1α to ER stress, then induction of BiP (or any process downstream of XBP1s that ameliorates ER stress) could block activation of IRE1α while leaving PERK apparently unaffected. This would occur if the concentration of stressor used were to be above the threshold required to activate PERK but below that required to activate IRE1α. To address this, ranges of DTT, tunicamycin, and thapsigargin concentrations could be tested for their effects on IRE1α and PERK, in the presence and absence of overexpressed XBP1s. If a selective negative feedback mechanism exists linking XBP1s to the inhibition of XBP1u splicing, then the EC50s of these agents will increase only for the activation of IRE1α.

**Minor concerns **

1. It is unclear why the phosphorylation of IRE1α has not been measured. This would help determine at what stage XBP1s antagonizes IRE1α signaling.
2. The kinetics of activation and deactivation of IRE1α and PERK differ significantly ^3^. Have these been examined in this system?
3. Does expression of XBP1s inhibit the activity of an IRE1α cytosolic domain activated independently of ER stress, e.g. the IRE1 constructs described in refs ^3^^,^^8^?

I have read this submission. I believe that I have an appropriate level of expertise to confirm that it is of an acceptable scientific standard, however I have significant reservations, as outlined above.

## References

[1] RonDWalterP: Signal integration in the endoplasmic reticulum unfolded protein response. *Nat Rev Mol Cell Biol*.2007;8(7) : 10.1038/nrm2199 519-29 10.1038/nrm2199 17565364

[2] WalterFSchmidJDüssmannHConcannonCGPrehnJH: Imaging of single cell responses to ER stress indicates that the relative dynamics of IRE1/XBP1 and PERK/ATF4 signalling rather than a switch between signalling branches determine cell survival. *Cell Death Differ*.2015;22(9) : 10.1038/cdd.2014.241 1502-16 10.1038/cdd.2014.241 25633195PMC4532775

[3] LinJHLiHYasumuraDCohenHRZhangCPanningBShokatKMLavailMMWalterP: IRE1 signaling affects cell fate during the unfolded protein response. *Science*.2007;318(5852) : 10.1126/science.1146361 944-9 10.1126/science.1146361 17991856PMC3670588

[4] YoshidaHMatsuiTYamamotoAOkadaTMoriK: XBP1 mRNA is induced by ATF6 and spliced by IRE1 in response to ER stress to produce a highly active transcription factor. *Cell*.2001;107(7) :881-91 1177946410.1016/s0092-8674(01)00611-0

[5] CalfonMZengHUranoFTillJHHubbardSRHardingHPClarkSGRonD: IRE1 couples endoplasmic reticulum load to secretory capacity by processing the XBP-1 mRNA. *Nature*.2002;415(6867) : 10.1038/415092a 92-6 10.1038/415092a 11780124

[6] LeeKTirasophonWShenXMichalakMPrywesROkadaTYoshidaHMoriKKaufmanRJ: IRE1-mediated unconventional mRNA splicing and S2P-mediated ATF6 cleavage merge to regulate XBP1 in signaling the unfolded protein response. *Genes Dev*.2002;16(4) : 10.1101/gad.964702 452-66 10.1101/gad.964702 11850408PMC155339

[7] BertolottiAZhangYHendershotLMHardingHPRonD: Dynamic interaction of BiP and ER stress transducers in the unfolded-protein response. *Nat Cell Biol*.2000;2(6) : 10.1038/35014014 326-32 10.1038/35014014 10854322

[8] HanDUptonJPHagenACallahanJOakesSAPapaFR: A kinase inhibitor activates the IRE1alpha RNase to confer cytoprotection against ER stress. *Biochem Biophys Res Commun*.2008;365(4) : 10.1016/j.bbrc.2007.11.040 777-83 10.1016/j.bbrc.2007.11.040 18035051

# Response

**Published:** 2017-09-28

**Authors:** Neil Bulleid

**Affiliations:** University of Glasgow, UK

The main concern of this reviewer was that the results could be explained if the sensitivity of Ire1 or PERK to UPR induction was different so that, at the concentrations we are using, there could be a differential effect. We carried out the experiment suggested by the reveiwer, i.e. a titration of the response to tunicamycin in the absence or presence of exogenous XBP1. We include the result as a new figure in our revised version (Figure 5). Interestingly the result demonstrates that the exogenous XBP1 suppresses Ire1 splicing activity but does not alter its sensitivity towards the inducer. The conclusion is that Ire1 can still be activated but its activity is in some way inhibited by the presence of exogenous XBP1. No effect was seen for PERK which showed the same sensitivity to the UPR inducer in the presence or absence of exogenous XBP1.

The minor concerns of the reviewer can be addressed as follows:

1. We have tried several commercial antibodies to IRE1α but none recognise the hamster protein. We can indirectly conclude that Ire1α is activated from the splicing assays carried out but cannot assay its phosphorylation status.
2. We have not carried out an analysis of the kinetics of activation or inactivation.  As our comparison is between the absence of presence of exogenous XBP1, we focused on the difference between the splicing activity towards endogenous XBP1 in this paper. 
3. We have not carried out the experiments with the cytosolic domains of Ire1α suggested by the reviewer.  We do know that in the absence of an exogenous agent to activate the UPR we still see a supression of XBP1 splicing in the presence of exogenous XBP1. This is likely due to a low level of ER stress that occurs even in the absence of external agents. 
